# Selective and Accurate Determination Method of Propofol in Human Plasma by Mixed-Mode Cation Exchange Cartridge and GC-MS

**DOI:** 10.1155/2016/9531769

**Published:** 2016-08-11

**Authors:** Jae Sung Pyo

**Affiliations:** College of Pharmacy, Kyungsung University, Busan 48434, Republic of Korea

## Abstract

A gas chromatography-mass spectrometry (GC-MS) method for the determination of propofol in human plasma has been developed and validated. Propofol was extracted from human plasma by using mixed-mode cation exchange/reversed-phase (MCX) cartridges. As propofol easily volatilizes during concentration, 100% methanol was injected directly into GC-MS to elute propofol. Despite avoiding concentration process of the eluted solution, lower limit of quantization (LLOQ) of propofol was 25 ng/mL. The validated method exhibited good linearity (*R* ^2^ = 0.9989) with accuracy and precision −5.8%~11.7% and 3.7%~11.6%, respectively. The other validation parameters, recovery and matrix effect, ranged from 96.6% to 99.4% and 95.3% to 101.4%, respectively. Propofol standard was quantified to evaluate possible loss due to the concentration processes, nitrogen gas and centrifugal vacuum. These two concentration processes resulted in notable decrease in the quantity of propofol, signifying avoiding any concentration processes during propofol quantification. Also, to confirm suitability of the developed method, authentic human plasma samples were analyzed. The selective assay method using MCX cartridge and GC-MS facilitated quantification of propofol in plasma sample accurately by preventing any losses due to the concentration processes.

## 1. Introduction

Propofol (2,6-diisopropylphenol) is an intravenously administered hypnotic/amnestic agent for induction and maintenance of anesthesia. A therapeutic dose of intravenous propofol produces hypnosis rapidly and smoothly within forty seconds [1–3]. Because of its short and rapid effects, propofol has been most preferred for anesthesia. Since 1980, it was also considered safe. In 2000, propofol was preferred anesthetic agent for inducting anesthesia in 96.5% of urology and orthopedic day-surgeries in the UK and for about 50% of cardiac surgeries in France [4].

However, high lipophilicity of propofol can cause hypertriglyceridemia and bradycardia, subsequent hypotension, and transient apnea at high dose infusion [1, 5]. Also, other adverse reactions reported include fever, cardiac arrhythmia, somnolence, and seizures. After the death of Michael Jackson, toxicity and abuse of propofol have attained abrupt attention at local and international levels. Long term use of propofol infusions among children has resulted in fatal cardiac arrest in at least five cases [6, 7]. In South Korea, 33 fatal cases related to propofol administration were reported during 2000–2010, 16 medical accidents and 17 suicidal and accidental deaths of hospital employees [8]. Because of such abuse and accidents, propofol was classified as a controlled drug in South Korea, since 2011.

Many studies for propofol determination were conducted using gas chromatography-mass spectrometry (GC-MS) and liquid chromatography-tandem mass spectrometry (LC-MS/MS). Propofol and 3 main metabolites (propofol-glucuronide, 1-quinol-glucuronide, and 4-quinol-glucuronide) in human plasma were extracted by using the solid phase extraction and validated with the LC-MS/MS [9]. GC-MS and LC-MS/MS methods for the analysis of propofol and its main metabolites in human urine were compared, and some of the metabolites were derivative and identified with GC-MS [10].

In the propofol analysis that used LC-MS, derivatization or restricted mass condition for propofol determination should be embraced because of the low ionization efficiency of propofol in LC-MS/MS. The absence of ionization group and nonpolarity of propofol cause such low ionization efficiency. To overcome this hindrance, N-methylpyridinium ether derivatization and dansyl chloride derivatization were applied to the propofol analysis [11, 12]. Azo coupling with LC-MS/MS was employed to enhance the sensitivity of propofol [13]. Silylation with GC-MS and azo coupling with LC-MS/MS were compared for propofol determination in blood [14]. These derivatization methods focused on propofol sensitivity in spite of inconvenient sample preparation and less precision and accuracy.

The therapeutic dose of propofol is quite high (6–10 mg/L for induction of anesthesia and 2–4 mg/L for maintenance of anesthesia) [11]. The concentration of propofol in plasma indicates the cause of death related to medical malpractices and suggests an important evidence for propofol accidents in the forensic field. Therefore, propofol quantification methods should concentrate on accuracy, precision, and recovery rather than LLOQ. However, many forensic laboratories and studies have focused on enhancing the LLOQ through concentration which could result in propofol volatilization during solvent concentration [15]. Derivatization steps may also induce inaccurate quantitation results because of complicated steps and low reproducibility of derivatization. Accordingly, propofol analysis should be coupled with nonvolatile extraction. In the same vein, extraction solvent containing acidifying or alkalifying agent [11] could not be employed for propofol extraction because of incompatibility with GC-MS.

In this experiment, mixed-mode cation exchanger with two binding sites, sulfate ion site for ionic interaction and benzene ring site for reversed-phase interaction, was applied to extract propofol from human plasma. Also, propofol volatility was evaluated using two concentrators: nitrogen gas and centrifugal vacuum. This is a simple, rapid, and accurate quantification method to determine the propofol in human plasma, without sample concentration. It could be a new guideline for propofol analysis in pharmaceutical and/or in forensic fields.

## 2. Methods and Materials

### 2.1. Reagents and Chemicals

Cannabinol (CBN), used as internal standard, and propofol were purchased from Sigma (St. Louis, MO, USA). Methanol was supplied by Burdick and Jackson (Muskegon, MI, USA). Water was purified using a Millipore (Chem-science, USA) purification system. Oasis MCX® (3 mL, 60 mg) cartridges were purchased from Waters (Milford, MA, USA).

### 2.2. Sample Preparation

200 *μ*L of blood sample, 1 mL of phosphate buffer saline (PBS) solution, 50 *μ*L of the internal standard (1.0 mg/L), and 50 *μ*L of calibration standards (0.1, 0.2, 1, 2, 4, 10, and 20 mg/L) were transferred to 1.5 mL micro tube. Then the micro tube was sonicated for 15 min and was centrifuged for 10 min at 12,000 ×g to remove any undissolved residue.

### 2.3. Solid Phase Extraction

The SPE cartridges (Oasis MCX) were preconditioned with 2 mL methanol and 2 mL distilled water, sequentially. After being preconditioned, the centrifuged plasma sample was then loaded into the cartridges, previously washed with 2 mL distilled water and 2 mL cyclohexane, and dried under reduced pressure for 5 min. Elution of analytes was carried out with 2 mL of methanol. 100 *μ*L of eluted methanol was filtered with 0.22 *μ*m PVDF microporous membrane (Millipore, Billerica, MA) and introduced into 200 *μ*L vials. Finally, 1 *μ*L of final solution was injected into the GC-MS.

### 2.4. GC-MS Conditions

The GC-MS analysis was performed with a 7890A gas chromatography instrument, combined with 5975C mass spectrometer equipped with electron ionization (EI) and quadrupole analyzer (Agilent technologies, Palo Alto, CA, USA).

Propofol was separated using a 100% dimethyl polysiloxane fused-silica capillary column (HP-5MS 30 m × 250 *μ*m I.d. film thickness 1.00 *μ*m, Agilent technologies, Palo Alto, CA, USA). The ion source and interface temperatures were set at 230°C and 250°C, respectively. A splitless injection mode was employed, at 250°C. The mass scan range was from 50 to 550 amu. The electron energy was set at 70 eV. The oven temperature was programmed to hold at 80°C for 5 min and then increase to 290°C at the rate of 20°C/min.

### 2.5. Calibration Curve and Validation Procedure

The analytical method was validated in terms of the limit of detection, the limit of quantification, linearity, intra- and interday precision and accuracy, recovery, and the matrix effect. Propofol free plasma samples from five different volunteers were used as blanks, and various concentrations of propofol standards were spiked to the samples. To evaluate the selectivity of the method, six different sources of blank plasma samples were extracted and analyzed to check interfering peak on propofol and the internal standard. Recovery was assessed to compare the concentration of spiking propofol, at three different concentrations, between, before, and after extraction. Propofol spiked plasma from six different sources was compared with propofol calibrator spiked phosphate buffer saline to achieve the matrix effect.

The linearity of calibrator was calculated by analyzing five calibration curves, ranging from 25 to 5,000 ng/mL. The limit of detection (LOD) and the lower limit of quantification (LLOQ) were assessed, using signal to noise ratio. The signal to noise ratios of LOD and LLOQ were 3 and 10, respectively. Accuracy and precision were achieved by analyzing propofol spiked plasma at three different concentrations (25, 500, and 5,000 ng/mL) and were replicated five times. In order to examine interday precision and accuracy, five sets of each sample were analyzed on three different days.

### 2.6. Analysis of Authentic Plasma Samples

Two blood samples were obtained from the police for forensic analysis. These samples were stored at 4°C in refrigerator and were used for this (validated) method. Propofol concentration was achieved using peak area and calibration curve.

### 2.7. Volatility of Propofol

#### 2.7.1. Nitrogen Gas Evaporation

The nitrogen gas evaporation was performed at an EvaT-0200 total concentration system (Goojung, Seoul, South Korea), with the temperature of nitrogen gas concentrator set at 45°C and the pressure of nitrogen gas at 20 psi. 100 *μ*L of propofol (10 ng/mL) was mixed with known volume of methanol (0 *μ*L, 100 *μ*L, 400 *μ*L, 900 *μ*L, and 1900 *μ*L) in five different glass tubes. After mixing, the glass tubes were evaporated until dried under a gentle stream of nitrogen. The glass tubes were taken out as soon as possible after the evaporation of the solvent. The dry residue was reconstituted with 100 *μ*L of cannabinol (10 ng/mL) and 1 *μ*L of solution was injected into the GC-MS. All the processes were triplicated and calibrated.

### 2.8. Centrifugal Vacuum Concentration

Centrifugal vacuum concentration system consisted of a UNIVAPO 100H vacuum concentrator centrifuge and a UNIJET II refrigerated aspirator (UNIEQUIP, Munich, Germany). The temperatures of the vacuum concentrator centrifuge and the refrigerated aspirator were set at 40 and 7°C, respectively. The pressure of the refrigerated aspirator was 145 psi.

100 *μ*L of propofol (10 ng/mL) was mixed with the volume of methanol (0 *μ*L, 100 *μ*L, 400 *μ*L, 900 *μ*L, and 1900 *μ*L) in five different micro tubes, vortexed momentarily and concentrated in vacuum using the centrifugal vacuum evaporator. Each micro tube was taken out as soon as possible after solvent had evaporated. The dry residue was reconstituted with 100 *μ*L of cannabinol (10 ng/mL) and 1 *μ*L of solution was injected into the GC-MS. All the processes were triplicated and calibrated.

## 3. Results and Discussion

### 3.1. Sample Extraction Using MCX Cartridge

The MCX cartridge has two binding sites. The first is the benzene ring site binding to the benzene ring of the target analyte with *π*-*π* bonding, while the other is negatively charged sulfur trioxide site combining to the positively charged part of the analyte.

While propofol was loaded to the mixed-mode cation cartridge with phosphate buffer (pH 6.8), propofol was in neutral form and not in ionic condition because p*K*_*a*_ value of propofol is 11.1. Therefore, propofol could only bind to the benzene ring of cartridge sorbent by reversed-phase retention. The compounds which could bind to neither the benzene ring nor the negative site of sorbent were eliminated through the water washing step. Sequential cyclohexane washing step removed only the nonpolar interferences. The cyclohexane washed solution was analyzed with GC-MS and did not show any peaks. This confirmed that there were no traces of propofol in the washing solution and propofol was still bound to the sorbent after the cyclohexane washing procedure.

**Figure 1:**
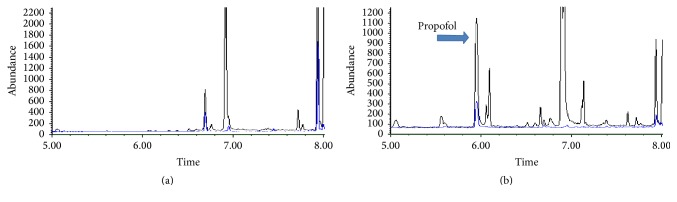
The extracted ion chromatograms of blank sample (a) and propofol spiked plasma sample (b).

After the washing steps, some chemicals like propofol bound to the benzene ring of cartridge sorbent with reversed-phase retention. Other analytes with cation group binding to the negatively charged site of sorbent with ionic binding were not eluted through water and cyclohexane washing. Among the combined materials, only chemicals including propofol bound to the cartridge, with the reversed-phase retention being extracted at the methanol elution step (Figure 1(b)). During this elution process, binding between the chemicals and cation sorbent of cartridge was not affected by methanol elution. Rather, it is maintained, because the strength of methanol was not sufficient to disconnect the ionic binding. Through these washing and elution steps propofol was selectively and clearly eluted by methanol.

### 3.2. Method Validation

The propofol standards at seven concentration levels, 25, 50, 250, 500, 1,000, 2,500, and 5,000 ng/mL, were used for the method validation. The linearity of 5 calibration curves was drawn, and their correlation coefficients (*R* ^2^) were 0.9989 with mean slope and intercept 0.1663 and 0.7098, respectively. The LOD and LLOQ of propofol were 10 ng/mL and 25 ng/mL, respectively. The LLOQ value was obtained by assessing the signal to noise ratio. However, the LLOQ value was 200 times as low as therapeutic level. The results of the intra- and interday precision and accuracy were within 11.6% for precision and 11.7% for accuracy, as shown in Table 1. The recoveries and matrix effect of three concentrations of propofol have been summarized in Tables 2 and 3, respectively. The mean values of recoveries were over 96.6%, which demonstrates the remarkable efficiency of this extraction method. The matrix effect ranged from 95.3 to 101.4% and the CV values of matrix effects are below 13.7%. These values indicate that the described extraction method may not be affected by endogenous matrix of plasma.

### 3.3. Authentic Human Plasma

**Figure 2:**
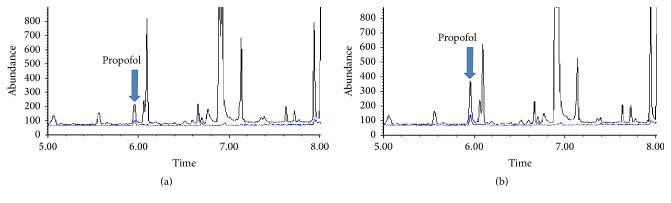
The representative extracted ion chromatograms of authentic heart plasma sample (a) and peripheral plasma sample (b).

Plasma samples of 2 patients were extracted according to this developed method. The propofol concentrations of heart blood and peripheral blood were determined and calculated. Their representative chromatograms are shown in Figure 2 and the quantification results are summarized in Table 4.

### 3.4. Volatility of Propofol

GC-MS analysis was used to evaluate the loss of propofol by nitrogen gas concentration and centrifugal vacuum evaporation. Various volumes of sample but with equal quantity of propofol were concentrated with nitrogen gas evaporator and centrifugal vacuum evaporator and quantified with GC-MS. However, regardless of concentration methods, all the results showed loss of propofol, and the inconsistent quantification results were turned up (Table 5). Moreover, huge variation in their standard deviations indicates low reproducibility. This uneducable propofol loss necessarily induces inaccurate propofol quantification leading to imprecise cause of deaths, in the forensic field. Many researchers looked past the volatile characteristic of propofol, the boiling point of propofol being 256°C at 764 mmHg. Accordingly, the propofol samples were concentrated for increasing concentration or elimination of buffer and water, before GC-MS analysis. In this research, propofol was eluted with 100% methanol, and the eluted solution was directly injected to GC-MS.

## 4. Conclusions

The nitrogen gas concentration and centrifugal evaporation tests suggested propofol to be a very volatile compound, indicating that the general propofol quantification methods with nitrogen gas concentration result in inaccurate quantification value. However, in this study, propofol was extracted from human plasma with mixed-mode cartridge, stably without any loss due to concentration. Also, this simple and accurate method was validated and the results were satisfactory. Furthermore, authentic human plasma samples were successfully applied to the method. Thus, this extraction method could be a new guideline for other researchers and can be employed in forensic and other analytical fields.

## Figures and Tables

**Table 1:** Intra- and interday precision and accuracy of propofol.

Concentration (ng/mL)	Intra-assay		Interassay	
	Precision (CV%)	Accuracy (bias, %)	Precision (CV%)	Accuracy (bias, %)
25	5.4	5.3	11.6	11.7
500	4.0	−4.2	5.4	−5.8
5,000	3.7	−0.1	4.1	−1.2

**Table 2:** Recovery of propofol in human plasma (*n* = 6).

Concentration (ng/mL)	Recovery (%)	CV value of recovery
25	96.6 ± 3.3	9.1
500	98.8 ± 2.2	5.3
5,000	99.4 ± 0.9	2.3

All the concentrations in mg/L of plasma; mean values ± standard error.

**Table 3:** Matrix effect of propofol in human plasma (*n* = 6).

Concentration (ng/mL)	Matrix effect (%)	CV value of matrix effect
25	95.3 ± 5.9	13.7
500	100.2 ± 2.5	6.2
5,000	101.4 ± 0.3	0.1

All the concentrations in mg/L of plasma; mean values ± standard error.

**Table 4:** Propofol concentration of authentic human heart blood and peripheral blood.

Concentration (mg/L)	Case 1	Case 2
Heart blood	0.238	0.454
Peripheral blood	0.988	0.997

**Table 5:** The quantitation results of concentrated propofol standards with two different kinds of evaporation.

Added methanol volume (*μ*L)	0	100	400	900	1900
Recovery (%) of nitrogen gas evaporation	100 ± 1.3	15.4 ± 4.0	10.6 ± 2.0	6.5 ± 1.5	4.9 ± 1.4
Recovery (%) of centrifugal vacuum evaporation	100 ± 3.0	10.6 ± 2.2	6.1 ± 1.1	3.3 ± 0.6	3.1 ± 0.7

Recovery was calculated by dividing the area of 0 *μ*L methanol added propofol sample.

All the recoveries are displayed with mean values ± standard error.

## References

[1] Kotani Y., Shimazawa M., Yoshimura S., Iwama T., Hara H. (2008). The experimental and clinical pharmacology of propofol, an anesthetic agent with neuroprotective properties. *CNS Neuroscience and Therapeutics*.

[2] Levy R. J. (2011). Clinical effects and lethal and forensic aspects of propofol. *Journal of Forensic Sciences*.

[3] Wilson C., Canning P., Caravati E. M. (2010). The abuse potential of propofol. *Clinical Toxicology*.

[4] Roussin A., Montastruc J.-L., Lapeyre-Mestre M. (2007). Pharmacological and clinical evidences on the potential for abuse and dependence of propofol: a review of the literature. *Fundamental and Clinical Pharmacology*.

[5] Teshima D., Nagahama H., Makino K., Kataoka Y., Oishi R. (2001). Microanalysis of propofol in human serum by semi-microcolumn high-performance liquid chromatography with UV detection and solid-phase extraction. *Journal of Clinical Pharmacy and Therapeutics*.

[6] Burow B. K., Johnson M. E., Packer D. L. (2004). Metabolic acidosis associated with propofol in the absence of other causative factors. *Anesthesiology*.

[7] Salengros J.-C., Velghe-Lenelle C.-E., Bollens R., Engelman E., Barvais L. (2004). Lactic acidosis during propofol-remifentanil anesthesia in an adult. *Anesthesiology*.

[8] Seo J. (2011). Forensic review of propofol induced deaths—analysis of 33 autopsied cases. *Annual Report National Forensic Service*.

[9] Cohen S., Lhuillier F., Mouloua Y., Vignal B., Favetta P., Guitton J. (2007). Quantitative measurement of propofol and in main glucuroconjugate metabolites in human plasma using solid phase extraction-liquid chromatography-tandem mass spectrometry. *Journal of Chromatography B*.

[10] Lee S. Y., Park N.-H., Jeong E.-K. (2012). Comparison of GC/MS and LC/MS methods for the analysis of propofol and its metabolites in urine. *Journal of Chromatography B*.

[11] Thieme D., Sachs H., Schelling G., Hornuss C. (2009). Formation of the N-methylpyridinium ether derivative of propofol to improve sensitivity, specificity and reproducibility of its detection in blood by liquid chromatography-mass spectrometry. *Journal of Chromatography B: Analytical Technologies in the Biomedical and Life Sciences*.

[12] Beaudry F., Guénette S. A., Winterborn A., Marier J.-F., Vachon P. (2005). Development of a rapid and sensitive LC-ESI/MS/MS assay for the quantification of propofol using a simple off-line dansyl chloride derivatization reaction to enhance signal intensity. *Journal of Pharmaceutical and Biomedical Analysis*.

[13] Vaiano F., Mari F., Busardò F. P., Bertol E. (2014). Enhancing the sensitivity of the LC-MS/MS detection of propofol in urine and blood by azo-coupling derivatization. *Analytical and Bioanalytical Chemistry*.

[14] Vaiano F., Serpelloni G., Focardi M., Fioravanti A., Mari F., Bertol E. (2015). LC-MS/MS and GC-MS methods in propofol detection: evaluation of the two analytical procedures. *Forensic Science International*.

[15] Han E. Y. (2014). A study of analytical methods for the determination of propofol in blood. *Archives of Pharmacal Research*.

